# The Role of Insulin-like Growth Factor (IGF) System in the Corneal Epithelium Homeostasis—From Limbal Epithelial Stem Cells to Therapeutic Applications

**DOI:** 10.3390/biology13030144

**Published:** 2024-02-25

**Authors:** Małgorzata Woronkowicz, Harry Roberts, Piotr Skopiński

**Affiliations:** 1 NDDH, Royal Devon University Healthcare NHS Foundation Trust, Barnstaple EX31 4JB, UK; 2 Moorfields Eye Hospital NHS Foundation Trust, 162 City Road, London EC1V 2PD, UK; 3 West of England Eye Unit, Royal Devon University Healthcare NHS Foundation Trust, Exeter EX2 5DW, UK; harry.roberts@nhs.net; 4 University of Exeter Medical School, Exeter EX1 2HZ, UK; 5 Department of Ophthalmology, SPKSO Ophthalmic University Hospital, Medical University of Warsaw, 00-576 Warsaw, Poland; pskopin@wp.pl; 6 Department of Histology and Embryology, Medical University of Warsaw, 02-004 Warsaw, Poland

**Keywords:** limbal epithelial stem cells, insulin-like growth factor system, insulin, IGF-1, IGF-2, IGFBP, corneal epithelium, diabetes

## Abstract

**Simple Summary:**

The corneal epithelium is a protective barrier and refractive structure in the eye maintained through a complex regenerative process involving the lacrimal gland, tear film, and corneal nerves. This review explores the insulin-like growth factor (IGF) system and its role in corneal epithelium homeostasis. Emphasis is placed on the significance of limbal epithelial stem cells and potential therapeutic applications targeting the system components.

**Abstract:**

The corneal epithelium, comprising three layers of cells, represents the outermost portion of the eye and functions as a vital protective barrier while concurrently serving as a critical refractive structure. Maintaining its homeostasis involves a complex regenerative process facilitated by the functions of the lacrimal gland, tear film, and corneal nerves. Crucially, limbal epithelial stem cells located in the limbus (transitional zone between the cornea and the conjunctiva) are instrumental for the corneal epithelium integrity by replenishing and renewing cells. Re-epithelialization failure results in persistent defects, often associated with various ocular conditions including diabetic keratopathy. The insulin-like growth factor (IGF) system is a sophisticated network of insulin and other proteins essential for numerous physiological processes. This review examines its role in maintaining the corneal epithelium homeostasis, with a special focus on the interplay with corneal limbal stem cells and the potential therapeutic applications of the system components.

## 1. Introduction

The corneal epithelium is the outermost part of the cornea and consists of three cellular layers: the superficial layer, middle wing layer, and the innermost basal cell layer with Bowman’s membrane in humans separating it from the corneal stroma [1]. It plays the role of a protective barrier as well as a refractive structure due to its avascular character. Its homeostasis is maintained through a complex regenerative process which takes about 10 days and involves proliferation and migration of epithelial cells [2]. In addition, the lacrimal gland function, tear film, and corneal nerves are pivotal in maintaining the health and integrity of the corneal epithelium [3].

The XYZ hypothesis, as proposed by Thoft and Friend, describes three phases of epithelial recovery, which include proliferation and stratification of limbal basal cells, their subsequent centripetal migration, and finally, desquamation of superficial cells [4]. Notably, in some mammalian species, the entire ocular surface serves as a source of epithelial stem cells [5]. A study by Chang et al. further suggests that human epithelial cells in the central cornea can also contribute to wound healing [6]. However, the failure of re-epithelialization may result in persistent corneal epithelial defects, which can be caused by limbal stem cell deficiency and keratopathy related to corneal exposure, surgical and non-surgical injuries, prior infections, diabetes, and neurotrophic and dry eye changes [7].

Limbal epithelial stem cells (LESCs) are instrumental in maintaining a healthy epithelium by continuously replenishing damaged and aging cells [8]. The limbus is a 1–2 mm transitional zone that separates the epithelium from the conjunctiva and constitutes a niche for LESCs [9]. This region provides a barrier, preventing the conjunctiva from invading the cornea and, as a consequence, reducing its transparency due to conjunctivalization. In a study from 1945, Mann indirectly demonstrated the presence of LESCs by describing the migration of pigmented basal cells in rabbit corneas [10]. Typically, they divide in an asymmetric pattern, generating two cells: a new LESC, which maintains its renewal capacity, and an early transient amplifying cell undergoing further differentiation and centripetal migration [8]. Recent studies suggest that LESC populations may vary in terms of their activity (active and quiescent pools), distribution in the limbus (outer vs. inner limbus), and roles in regenerative processes [11,12,13,14]. Unlike in the mouse limbus with a uniform LESC distribution [15], in humans, LESCs reside in the basal layer of numerous fibrovascular ridges, termed the Palisades of Vogt, and other tangentially and circumferentially extending structures such as crypts and pits [16,17]. This arrangement provides protection from the external environment and reduces the risk of damage from detrimental factors, such as ultraviolet radiation, chemicals, thermal burns, and pathogens [18]. At the same time, the LESC niche creates a microenvironment enabling interactions with biochemical mediators, including growth factors, cytokines, and chemokines.

**Figure 1:**
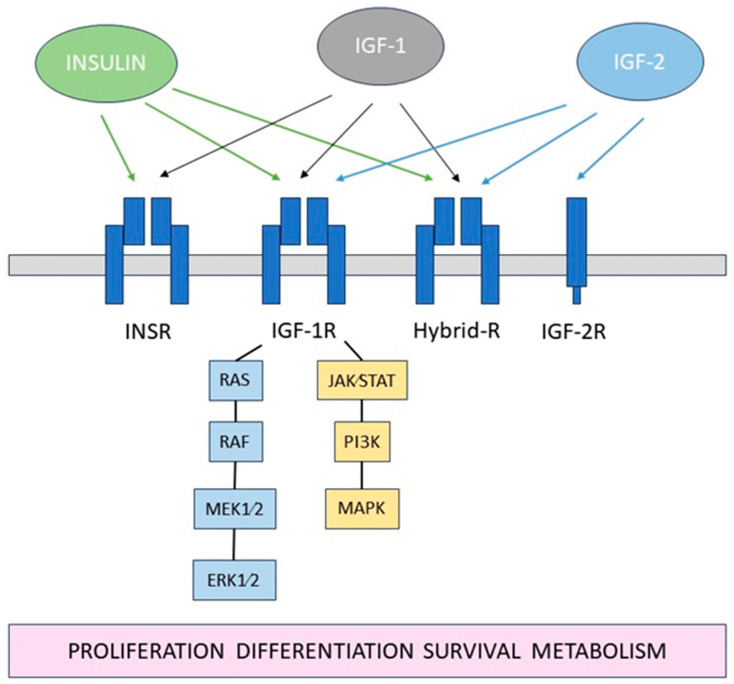
Schematic diagram of the IGF system.

The insulin-like growth factor (IGF) system is a complex network of hormones and proteins that play crucial roles in cell growth, development, and metabolism [19]. It consists of insulin, insulin-like growth factor 1 and -2 (IGF-1, IGF-2), their receptors: insulin receptor (INSR), IGF type 1 and 2 receptors (IGF-1R, IGF-2R), as well as several IGF-binding proteins (IGFBPs), Figure 1. 

Dysregulation of this system has been implicated in various diseases including diabetes, which profoundly impacts the eye. The IGF system is also pivotal for corneal epithelium homeostasis, influencing critical cellular processes and potentially serving as a target in therapeutic applications (Table 1).

## 2. The Role of Insulin and Effect of Diabetes

Insulin is a polypeptide hormone consisting of two A and two B chains, produced by pancreatic beta cells and secreted in response to a high blood glucose level [19]. It has metabolic effects and plays a role in various phases of the cell cycle, from growth to apoptosis. The presence of INSR in the cornea was first demonstrated by Naeser [41]. Alternative splicing of INSR occurs at exon 11, leading to the generation of two distinct isoforms: INSRA and INSRB [19]. An immunohistochemical analysis by Rocha et al. demonstrated that INSR is expressed in the corneal epithelium [42]. In their study of human corneas, INSR was predominantly localized within the cytoplasm and plasma membrane in the wing and superficial cell layers, with noticeable variability in its expression across the basal and intermediate suprabasal cells (Table 2).

Diabetes mellitus, a chronic metabolic disorder characterized by hyperglycemia, results from a deficiency in insulin secretion, impaired insulin action, or a combination of both [45]. The two primary forms of diabetes, type 1 and type 2, differ in their pathophysiology but share the common feature of dysregulated glucose metabolism. In type 1 diabetes, an autoimmune response leads to the destruction of pancreatic beta cells, resulting in insufficient insulin production, while type 2 diabetes involves a combination of insulin resistance and relative insulin deficiency. Diabetes can significantly impact the eye, including the ocular surface, leading to dysfunction of epithelium and development of diabetic keratopathy.

Unlike most tissues, where insulin stimulates glucose uptake through the glucose transporter-4 (GLUT4), corneal epithelium is insulin-independent [46]. Glucose uptake in epithelial cells occurs through constitutively active glucose transporters, GLUT1, which undergo upregulation in case of a high metabolic demand, such as wound healing, similarly in diabetic and non-diabetic corneas.

Culture studies with human corneal epithelial cells demonstrated that insulin induces phosphorylation of extracellular signal regulated kinase (ERK 1/2), PI3-kinase [20] and epidermal growth factor receptor (EGFR), thereby promoting cell migration and wound healing [21]. Interestingly, increased expression of PI3K pathway kinases occurs in canine corneal cells following insulin treatment, contrasting with the observed lack of analogous effects in vitro in human corneal cells [47]. Within the corneal epithelium, insulin influences PTEN-induced kinase 1 (PINK-1)-mediated mitophagy and the mitochondrial accumulation of insulin receptor (INSR). Interactions between INSR and the voltage-dependent anion channel-1 (VDAC1) prevent fragmentation and altered polarization of mitochondria, as well as facilitate PINK-1-mediated mitophagy [22]. In diabetic rats, the process of histone H3 acetylation is reduced in corneal epithelial cells, resulting in compacted chromatin organization in nuclei characterized by increased size and elevated DNA ploidy [48].

A study by Song et al. showed that insulin can normalize the circadian rhythm of corneal cell mitosis via five main clock genes (Clock, Bmal1, Per2, Cry1, and Rev-erbα) whose expression is affected in diabetes [23]. Moreover, innate-like lymphocytes, such as γ δ T-cells expressing chemotactic factor IL-17 for neutrophils and monocytes, were found to be recruited to the corneal limbus in a diurnal pattern. In diabetes, the limbal cell migration is increased, potentially leading to the inflammatory state delaying wound healing, but is restored upon systemic insulin administration.

Insulin is secreted from the lacrimal gland and present in the tear film at a mean concentration of 0.404 ± 0.129 ng/mL, which is reduced in fasted individuals and shows no difference related to gender [42]. In diabetes, its secretion is reduced due to damage to the lacrimal gland and reduced corneal sensation, caused by hyperglycemia and oxidative stress [49]. In rat models of diabetes, histological analysis showed an increased lipofuscin level and higher malonaldehyde as well as peroxidase activity in the lacrimal glands compared to healthy animals [50]. Insulin signaling in rat lacrimal glands becomes impaired in the fourth week of diabetes, with the lacrimal gland serving as an extra pancreatic source of insulin for at least 4–7 weeks [51,52].

Several studies reported ocular surface abnormalities in patients with diabetes, such as reduced tear breakup time, lower tears secretion, and increased level of inflammatory markers: NPY, STAT-5 ICAM-1, and TNF-α [53,54,55]. Clinically, diabetic ocular surface complications include reduced corneal sensitivity and delayed epithelialization leading to dry eye syndrome, punctate corneal epitheliopathy, recurrent erosions, persistent epithelial defects, and neurotrophic keratopathy [49,56]. On a histological level, reduction in basal epithelial cell density and size, and increased intercellular space as well as increased corneal epithelial basement membrane thickness and irregularity were demonstrated in diabetic corneas [57,58,59,60,61]. It is postulated that diabetic ocular changes might be in part explained by the dysregulation of a pathway involving the opioid growth factor (OGF), i.e., [Met5]- enkephalin binding to nuclear-associated receptor (OGFr) [62]. In diabetes, serum OGF levels are elevated, and insulin may affect the OGF-OGFr axis [56,63,64].

A fundamentally negative impact of diabetes on the corneal epithelium is closely linked to its effect on LESC functioning [24]. A significant reduction in Palisades of Vogt in all four limbus quadrants was demonstrated in patients with type 2 diabetes using in vivo confocal microscopy [65]. In the same study, a higher risk of stem cell damage was noted in those with a high-density lipoprotein, triglycerides, and total cholesterol level above 1.215 mmol/L, 1.59 mmol/L, and 4.75 mmol/L, respectively.

Immunohistochemistry analysis of corneas from diabetic patients revealed a reduction in putative limbal stem cell markers, including ATP-binding cassette superfamily G member 2 protein (ABCG2), N-cadherin, ΔNp63α, K15, K17, K19, and β1 integrin [28]. This decrease in marker expression was associated with lower immunoreactivity and a diminished number of detected cells, indicating potential depletion or dysfunction of LESCs. Moreover, the ex vivo diabetic limbus was characterized by irregular epithelial basement membrane and reduced expression of laminin γ3 and fibronectin.

In another study, a reduction in expression of putative stem cell markers K15 and ΔNp63α was demonstrated in limbal epithelial stem cell (LESC)-enriched cultures obtained from the corneoscleral rims in diabetic patients with changes reversed by adenoviral gene therapy [27]. In an animal model of type 2 diabetes, reduced expression of corneal stem/progenitor cell markers, including Hes1, Keratin15, and p75, was observed in mice corneas [25]. Similarly, in mice with type 1 diabetes, the expression of putative LESC markers K15, ∆Np63α, and glycoprotein hormone alpha-2 (GPHA2) was reduced in the limbus [26].

A study employing a new method of objective quantification of immunofluorescence, aiming to overcome limitations of manual grading, observed a reduction in the putative LESC marker K14 in the limbus of diabetic mice [24]. Furthermore, reduced expression of putative limbal epithelial stem cell markers, such as paired box protein-6 (PAX6), ∆Np63α, K15, K17, and membrane transporter ABCG2, was demonstrated in cultured diabetic human limbal epithelial cells, which was associated with slower corneal epithelial wound healing [29]. It is crucial to note, however, that several cell markers for LESCs can also be identified in other parts of the eye, and their use has limitations in discriminating pure stem cells from early transient amplifying cells [24,66].

The nervous system plays an instrumental role in maintaining the homeostasis of the corneal epithelium. Nerve branches of the sub-basal corneal nerve plexus are considered an important source of substances contributing to ocular surface health. Importantly, corneal innervation impacts a stem cells niche [67], with a limbal stem cells reduction reported following the destruction of the ophthalmic branch of the trigeminal nerve [25].

Detrimental effects of diabetes on corneal sensitivity, nerve fiber length, and density in both humans [68,69,70,71,72] and animals [25,73,74,75,76,77,78] have been reported in numerous studies and reviewed elsewhere [79,80,81]. Recently, a 3D tissue model of the human cornea was employed to demonstrate the degenerative effects of hyperglycemia on corneal nerves [82]. On a cellular level, pannexin1 channels present in corneal synaptosomes were found to be more glycosylated, characterized by enhanced membrane localization and leading to increased ATP release in diabetic subjects compared to non-diabetic controls [83]. Animal studies showed that insulin stimulates corneal nerve regeneration and expression of a limbal stem cell marker (DNp63) via Wnt signaling [84], and when applied topically, it exerts neuroprotective properties in diabetic rats [76]. In patients with type 2 diabetes, a nerve regenerative process can be limited by insulin resistance [85]. Interestingly, a prediabetic state is characterized by increased parameters of intra-epithelial corneal basal nerves, which could be attributed to the neurotrophic effect of higher insulin levels [78].

More recently, the overactivation of the ocular sympathetic nervous system adjacent to the limbus, mediated through the β2-adrenoceptor NE-Adrb2- sonic hedgehog (Shh) signaling pathway, was reported in type 1 diabetic mice [26]. This overactivation led to dysfunction and reduced proliferation of limbal stem/progenitor cells in response to a chemical injury. Additionally, the interplay between the nervous and immune system may impact limbal stem cells [24,86]. Immune cells residing in the cornea, such as T cells, interact with dendritic corneal cells and sensory nerves, influencing a response to acute and chronic stimuli [87]. A study with a mouse model of prediabetes demonstrated that dysfunction of corneal nerves, upregulation of inflammatory mediators, and reduction in neutrophil numbers in the limbus may precede a state of hyperglycemia [86]. In contrast, mechanical epithelium damage was associated with the accumulation of neutrophils in the limbus, possibly explained by reduced migratory capabilities of inflammatory cells, resulting in a slower healing response in diabetes.

MicroRNAs (miRNAs) are 18–25 nucleotides long non-coding RNAs that downregulate the expression of genes at a post-transcription level by binding complementary mRNAs [88]. They are involved in numerous cellular processes and can modulate multiple genes, making them a valuable research focus in regenerative medicine [89]. Studies suggest that they also play a role in LESC-associated processes, such as macropinocytosis, autophagy, and the expression of putative stem cell markers [90,91]. Funari at al. reported on the dysregulation of miRNA expression in human autopsy diabetic corneas, which was associated with abnormal wound healing [92]. Microarray analysis demonstrated that among the 29 miRNA studied, miR-146a, 21, and 424 were the most upregulated, while miR-509-3p and 143 were expressed at the lowest level. This is in line with other reports, which indicate that overexpression of miR-146a in the diabetic limbus may result in a reduced corneal inflammatory and healing response [24,93,94]. In another study, genome-wide sequencing was applied, identifying differences in expression profiles of 20 miRNA between normal and diabetic human corneas [95]. Results showed that miR-10b was upregulated in the diabetic limbus, with a higher increase observed in type 1 compared to type 2 diabetes. Moreover, altered expression of miRNA in exosomes have been demonstrated in diabetic limbal stem cells [96].

Overall, diabetes may affect the function of LESCs through various mechanisms and can potentially lead to severe, persistent corneal erosions. However, despite the detrimental effect on the cornea, the limbal barrier tends to be preserved, with no development of conjunctivalization or neovascularization characteristic for limbal stem cell deficiency [24].

Animal studies have demonstrated the beneficial effect of insulin treatment on epithelial wound healing in diabetic animals [97,98]. Klocek et al. found that corneal abrasions in type 1 diabetic mice, treated with topical insulin, reduced in size by 29% compared to the controls after 16 h [97]. Moreover, Zagon et al. reported a comparable rate of corneal healing in diabetic rats with an insulin implant and normal animals [98].

The first therapeutic use of topical insulin in humans was described by Aynsely in 1945 in a case series of patients with corneal ulcers [99]. Successful treatment with topical insulin for corneal conditions involving the epithelium has also been reported in subsequent studies summarized in Table 3.

Interestingly, a recent culture study with ocular surface cells showed that drops from plasma rich in growth factors were superior to topical insulin at two different concentrations (1 and 0.2 IU/m) in promoting wound healing and reducing the fibrosis process [115]. Currently, no universal protocol exists regarding treatment with topical insulin, as studies apply different preparation methods, dosages, concentrations, and types of drops [116,117].

Several studies have demonstrated novel ways of delivering insulin to the eye. A new delivery system, containing chitosan/poloxamer gel loaded with chitosan microparticles, has been reported to increase the local bioavailability of topical insulin in the treatment of the ocular surface in diabetic rats [118]. Additionally, electrospun fiber mats were shown to be effective in delivering insulin to the porcine cornea [119]. Recently, a convolutional neural network statistical analysis was applied to demonstrate the therapeutic effect of insulin liposomes on corneal epithelial defects in rats [120]. In a study of alkali-burned corneal models, a nano-system combining liposomes and trimethyl chitosan was used to deliver insulin and vascular endothelial growth factor small interfering RNA [121]. This combined therapy proved effective in reducing oxidative stress, increasing epithelialization, and inhibiting corneal neovascularization. Metabolomic analysis demonstrated that the therapeutic effect was possibly linked to insulin inhibiting the ferroptosis signaling pathway.

## 3. The Role of Insulin-like Growth Factor-1 (IGF-1) and -2 (IGF-2)

IGF-1 is a 7649 Daltons peptide hormone composed of seventy amino acids and produced mainly in the liver as a result of stimulation by human growth hormone [122]. IGF-1 binds to the IGF-1 receptor (IGF-1R) with 8 and 300 times higher affinity than IGF-2 and insulin, respectively [123]. Upon binding, it activates mitogen-activated protein kinase (MAPK) and extracellular signal-regulated kinase (ERK) [124]. IGF binding proteins (IGFBPs), which compete with IGF-1R, may prolong the half-life of free IGF-1 to several hours [125]. The concentration of IGF-1 in the tear film is comparable in males and females but declines with age [126]. Notably, the tear concentration of IGF-1 in diabetic mice was found to be reduced immediately after an alkaline chemical injury, as well as three and seven days later [127].

IGF-1 plays a significant role in cell growth, proliferation, and migration. A study involving a three-dimensional culture of human embryonic stem cells demonstrated that IGF-1 signaling is essential in the development of corneal epithelial and stromal cells, as evidenced by CK19 and vimentin markers [30]. Additionally, IGF-1 stimulates the expression of IGF receptors in limbal stem cells and promotes their differentiation into the epithelium [31]. This process is enhanced in the case of corneal injury, resulting in the downregulation of IGF-1R receptors in corneal cells and higher penetration of remaining IGF-1 into the limbal niche. Notably, IGF-1 was found to prevent the reduction in corneal stem/progenitor cells markers, such as Hes1, Keratin15, and p75, while also increasing nerve density in diabetic mice [25].

IGF-1 promotes the differentiation of murine mesenchymal stem cells into epithelia-like cells [128]. It is also speculated that the proliferation of human corneal epithelial cells facilitated by amniotic membrane occurs via IGF-1 [129]. Notably, corneal epithelial cells were demonstrated to release IGF-1, which in turn, increased the expression of N-cadherin (an adherens-junction protein) in corneal fibroblasts, enabling cell interactions to maintain tissue homeostasis [130]. IGF-1 has also been found to form complexes with vitronectin, resulting in enhanced corneal epithelium cell migration [32]. In addition, the migration of cultured human epithelial corneal cells is mediated by IGF-1 which increases the production of the matrix protein Ln-5 and its receptor β1 integrin via the PI3-K/AKT pathway [33]. Furthermore, it has been demonstrated that the protective effect of histatins (anti-microbial and anti-fungal proteins present in human saliva) in a cell model of UV-induced damage in human corneal epithelium occurs through the upregulated expression of IGF-1 and Bcl-2 [131].

IGF-1 treatment following laser in situ keratomileusis in rabbit eyes showed an association with a higher number of epithelial microvilli and a faster rate of nerve regeneration [132]. More recently, promising therapeutic outcomes were achieved by combining modified mRNA (modRNA) technologies with stem cells to treat corneas in mice subjected to alkali burns [133]. IGF-1 modRNA-engineered adipose-derived mesenchymal stem cells (ADSCs) showed superiority over normal ADSCs and IGF-1 protein eyedrops in promoting corneal epithelium healing, stimulating trigeminal ganglion cells activity, and maintaining stemness of limbal stem cells.

The IGF-1R is present in all layers of the cornea [122]. Its highest concentration has been observed in proximity to cellular nuclei within actively differentiating corneal epithelial cells, forming complexes with E-cadherins to augment cellular adhesion processes [43]. The IGF-1R and the INSR are transmembrane glycoproteins characterized by two extracellular alpha sub-units constituting the ligand-binding domain and two transmembrane beta subunits with inherent tyrosine kinase activity [134]. These receptors exhibit an amino acid sequence homology exceeding 50%. The shared structural features between the two receptors facilitate the formation of insulin and IGF-1 hybrid receptors (Hybrid-R). The precise mechanisms driving this formation remain elusive, with hypotheses suggesting a potential influence of the IGF-1R to INSR ratio or developmental factors. Moreover, Hybrid-R has been observed to exhibit a greater affinity for binding with IGF-1 compared than insulin, and its nuclear localization has been demonstrated in human corneal epithelial cells [44]. It is suggested that IGF-1R and INSR might be present in the nucleus of corneal epithelial cells only as Hybrid-R and as a result of upregulated expression of IGF-1R and INSR in the absence of insulin [44].

The synergistic effect of substance P (SP) and IGF-1 in promoting cell migration and, consequently, corneal epithelium healing, has been widely reported [135,136,137,138,139,140], mediated by p38 MAP kinase [141] and protein kinase C pathways [137]. Peptide FGLM-amide has been identified as the minimal component of SP capable of promoting corneal wound closure in combination with IGF-1 [139]. The combined action of SP and IGF-1 has proven effective in preventing superficial punctate keratopathy after cataract surgery in patients with diabetes [142] and treating persistent epithelial defects [143,144,145,146]. Moreover, this combination reduces epithelial healing time by 70 h on average in a rabbit eye after PRK [147]. When applied topically, SP (or the SP-derived peptide FGLM-amide) with IGF-1 improves corneal epithelial barrier function and enhances the healing process in rat models of neurotrophic keratopathy [148,149,150]. However, in dogs, no benefit of adding topical IGF-1 to SP was demonstrated when treating spontaneous chronic corneal epithelial defects [151].

Insulin-like growth factor-2 (IGF-2) and its receptor (IGF-2R) have been found in corneal epithelial cells and demonstrated to play a role in corneal regeneration [34,35]. Unlike insulin but similar to IGF-1, the IGF-2 structure has no D domain [152]. It binds to IGF-2R, which is a monomeric transmembrane protein comprising 15 different domains in its extracellular region [19]. Following injury, there is a significant increase in the expression of IGF-2 and its receptor in corneal peripheral cells. Moreover, IGF-2 has been shown to stimulate LESC differentiation, evidenced by the expression of K12 cell markers [34]. Pterygium, a common degenerative condition resulting in conjunctival overgrowth which may extend beyond the limbus and involve the cornea [153], was found to exhibit overexpression of IGF-2 and IGF-1R compared to normal conjunctiva, as revealed by immunohistochemical analysis [154].

## 4. The Role of IGF-Binding Proteins (IGFBPs)

The IGF-binding proteins (IGFBPs) regulate IGFs availability and activity, due to their equal or greater affinity than the IGF-1 receptor [125]. In the circulation, they increase the half-life of IGFs and block them from binding to the insulin receptor. While the IGFBP family members exhibit notable sequence homology, each possesses distinct structural features and functions.

IGFBP-2, a protein with a molecular weight of 36 kDa [155], plays a role in the growth and development of eye structures, with the highest ocular concentration measured in the cornea [36,156]. Its presence in the corneal germinal epithelium was demonstrated in developing rat eyes [37]. In chicken corneal epithelium, IGFB-2 was detected as early as at 3.5 days of embryonic development [36].

Insulin-like growth factor binding protein-3 (IGFBP3) is an N-linked glycosylated, phosphorylated, secretory protein [3] found in the cornea, including the cytoplasm of basal and suprabasal limbal epithelial cells [157]. It is a pleiotropic protein involved in cell survival by blocking IGF-1 from activating IGF-1R with hyperosmolar stress, reducing its expression [158,159]. IGFBP-3 acts as a molecular regulator of mitochondrial structure and function in epithelial cells [3]. The interplay between IGF-1R and IGFBP-3 contributes to corneal epithelium homeostasis, independently of P13K/Akt pathway [38]. Notably, IGFBP-3 promotes nuclear translocation of IGF-1R via SUMOylation by SUMO 2/3. Sirtuin (silent mating type information regulation 2 homolog) 1 (SIRT1), a class III histone deacetylase, inhibits IGFBP-3 via decreased acetylation of p53 which results in the activation of the IGF-1R/AKT pathway and promotes corneal epithelial wound healing [160]. IGFBP-3 was found in tears and at 2.8–3.5 higher concentration in diabetics compared to non-diabetic controls [161,162,163]. Moreover, in diabetes, its tear level shows a negative correlation with the length of nerve fibers and the density of nerve branches [161]. However, it is not fully clear if it is secondary to increased secretion or release from damaged corneal epithelium. In HSV-1 infected corneas, the immunofluorescence staining revealed the cytosolic accumulation and nuclear localization of IGFBP-3 within the infected corneal epithelial cells [164].

Insulin-like growth factor binding protein-5 (IGFBP-5) has been identified in the cornea, with upregulated expression demonstrated in diabetic rats [165]. A recent study showed that the expression of IGFBP-5 can be inhibited by microRNA miR-203 present in tears, resulting in reduced viability of corneal epithelial cells [39].

Insulin-like growth factor binding protein-7 (IGFBP-7) weighs 27 kDa and shows 94.4% similarity between human and mouse proteins [40]. It has high affinity for insulin and low affinity for IGF-1 as well as IGF-2 [166]. IGFBP-7 plays a role in angiogenesis, constitutes a target for transforming growth factor (TGF)-β1 [167], and is considered a biomarker of conjunctivalization in limbal stem cell deficiency [40].

## 5. Conclusions

In summary, the intricate interplay between the corneal epithelium and the surrounding microenvironment is essential for maintaining ocular health and function. Notably, the limbal epithelial stem cells, strategically located in the limbal region, serve as central players in ensuring the integrity of the corneal epithelium through their remarkable ability to replenish and renew cells.

The IGF system is involved in the regulation of corneal epithelial physiology, contributing to essential processes, such as wound healing, with implications for the activity of limbal epithelial stem cells. Insulin, particularly due to its pivotal association with diabetes, assumes a central role within the system. Diabetes represents a significant public health challenge, given its widespread prevalence and significant impact on the eye, including the corneal epithelium.

Further research is warranted to uncover the complexity of the interplay between the IGF system components, other signaling pathways, and the corneal epithelium. Elucidating the molecular mechanisms that govern their interactions will not only deepen our understanding of corneal epithelium homeostasis, but also pave the way for more targeted therapeutic interventions. In the future, advances in gene therapy and regenerative medicine involving limbal epithelial stem cells may offer promising avenues for manipulating these intricate processes to treat corneal disorders.

## Figures and Tables

**Table 1:** The overview of the key roles of the IGF system components in the corneal epithelium.

Component	Role	Study	Refs.
insulin	Promotes corneal epithelial cell migration and wound healing	In vitro with human cells	[20,21]
	Preserves mitochondrial function	In vitro with human cells	[22]
	Normalizes the circadian rhythm of corneal cells mitosis	In vivo in mice	[23]
	Promotes LESC proliferation and migration	Ex vivo with mice cornea	[24,25]
		In vivo in mice	[26]
		In vitro with human cells	[27]
		Ex vivo with human cornea	[28,29]
IGF-1	Promotes embryonic development	In vitro with human cells	[30]
	Stimulates differentiation of LESC	Ex vivo with mice cornea	[31]
	Enhances corneal epithelium cells migration	In vitro with human cells	[32,33]
IGF-2	Stimulates corneal regeneration	Ex vivo with mice and human cornea	[34,35]
	Promotes LESC differentiation	Ex vivo with mice cornea	[34]
IGFBPs	IGFBP-2 promotes embryonic development	Ex vivo with chick and rat cornea	[36,37]
	IGFBP-3 regulates mitochondrial structure and function	In vitro with human cells	[3]
	IGFBP-3 blocks IGF-1 from activating IGF-1R and promotes nuclear translocation of IGF-1R	In vitro with human cells	[38]
	IGFBP-5 increases viability of corneal epithelial cells	In vitro with human cells	[39]
	IGFBP-7 serves as biomarker of conjunctivalization	Ex vivo with human and mouse cornea	[40]

**Table 2:** Summary of the IGF system receptors and their localization in the corneal epithelium.

Receptor	Localization	Ref.
Insulin receptor	Plasma membrane and cytoplasm; mainly in the wing and superficial cell layers	[42]
	Nucleus	[43]
	Mitochondria	[22]
IGF-1R	All layers of the cornea; mainly around cellular nuclei of actively differentiating epithelial cells	[43]
	Plasma membrane and cytoplasm	[42]
	Mitochondria	[22]
Hybrid- R	Plasma membrane and nucleus	[44]
IGF-2R	Central and peripheral epithelium with higher expression in the periphery following corneal injury	[34]
	Primarily in the basal corneal epithelium in murine and porcine corneas	[35]

**Table 3:** Published studies evaluating results of a treatment of corneal conditions with epithelial defects with a topical insulin.

Ref.	Study Design	Diagnosis	No. of Eyes	Mean Age (Years)	Eyes with Complete Epithelialization (%)	Mean Time to Epithelialization (Days)
[100]	Randomized controlled trial	Postoperative corneal epithelial defect after vitreoretinal surgery in diabetics	A—8 B—8 C—8	A—62.62 ± 5.99 B—56.12 ± 7.77 C—55.75 ± 6.64	A—100 B—100 C—100	All eyes healed within 6 days 100% eyes in A, 75% eyes in B and 62.5% eyes in C group healed within 3 days.
[101]	Randomized clinical trial	Postoperative corneal epithelial defects after vitreoretinal surgery	19	57.05 ± 12.33	100	3
[102]	Prospective interventional, single-center case series	Refractory persistent epithelial defects	11	45.4 ± 25	82	62.3 ± 34.6
[103]	Prospective non-randomized hospital-based study	Refractory persistent epithelial defects	21	72.2	81	34.8 ± 29.9
[104]	Prospective non-randomized hospital-based study	Recurrent epithelial erosions	15	29.00 ± 8.72	100	21
[105]	Retrospective, observational	Refractory neurotrophic keratopathy (NK) in stages 2 and 3	21	61	90	18 ± 9 in NK stage 2; 29 ± 11 in NK stage 3
[106]	Retrospective, consecutive case–control series	Refractory persistent epithelial defects	61	71.5 ± 19.3	84	32.6 ± 28.3
[107]	Retrospective case series	Refractory neurotrophic corneal ulcers	6	36.5	100	7 to 25
[108]	Retrospective Case series	Corneal epithelial erosions induced during vitreoretinal surgery in diabetics	5	49	100	2.5 ± 0.6
[109]	Retrospective case series	Dry eye disease	32	61.3 ± 16.8	-	-
[110]	Case report	Corneal ulcer following chemical injury	1	41	100	60
[111]	Case report	Bilateral Neurotrophic keratitis	2	55	100	7
[112]	Case report	Neurotrophic keratopathy after resection of acoustic neuroma	1	45	100	14
[113]	Case report	Neurotrophic keratopathy	1	40	100	20
[114]	Case report	Neurotrophic keratopathy	1	64	100	30

A—eyes treated with topical insulin 0.5 unit QID, B—eyes treated with topical insulin 1 unit QID, C—eyes treated with topical insulin 2 units QID.

## Data Availability

No new data were created or analyzed in this study. Data sharing is not applicable to this article.
